# Supplementary figures and images for: Analysis of the Small Auxin-Up RNA (SAUR) Genes Regulating Root Growth Angle (RGA) in Apple
Source: Genes (Basel). 2022 Nov 15;13(11):2121. doi: 10.3390/genes13112121 (PMC9690538; doi:10.3390/genes13112121)

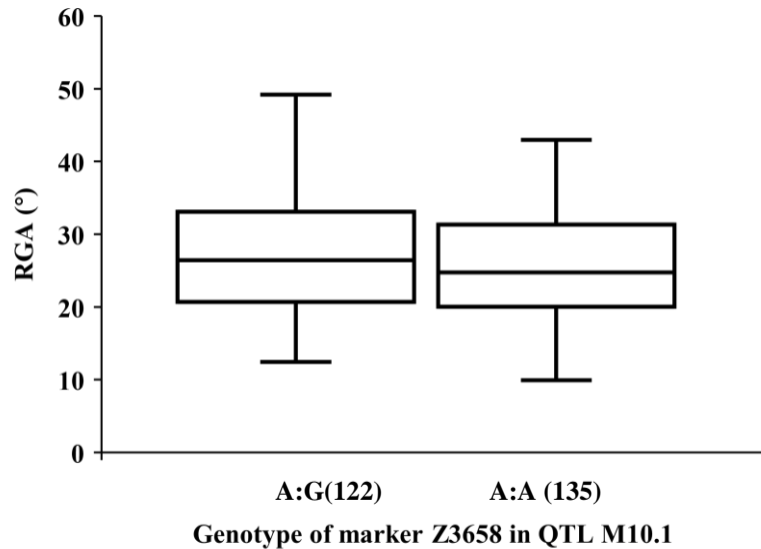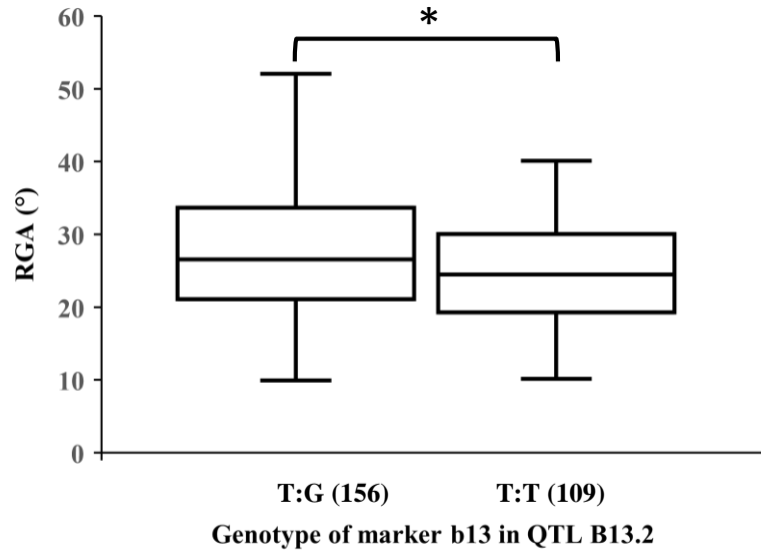

Supplement: Supplementary file 1 [file genes-13-02121-s001.zip › Figure S1-R.pdf]
